# Assessment of Telomere Length and Mitochondrial DNA Copy Number in Granulosa Cells as Predictors of Aneuploidy Rate in Young Patients

**DOI:** 10.3390/jcm11071824

**Published:** 2022-03-25

**Authors:** Tzu-Ning Yu, En-Hui Cheng, Han-Ni Tsai, Pin-Yao Lin, Chien-Hong Chen, Chun-Chia Huang, Tsung-Hsien Lee, Maw-Sheng Lee

**Affiliations:** 1 Institute of Medicine, Chung Shan Medical University, Taichung 40201, Taiwan; ningsyu@gmail.com; 2 Division of Infertility, Lee Women’s Hospital, Taichung 40652, Taiwan; enhuicheng@gmail.com (E.-H.C.); pin36.tw@yahoo.com.tw (H.-N.T.); jellylin0607@gmail.com (P.-Y.L.); clonemail@gmail.com (C.-H.C.); agarhuang@gmail.com (C.-C.H.); 3 Department of Obstetrics and Gynecology, Chung Shan Medical University Hospital, Taichung 40201, Taiwan

**Keywords:** ovarian aging, in vitro fertilization, pre-implantation genetic testing for aneuploidy, mitochondrial DNA copy number, telomere length

## Abstract

Background: To identify the correlation among female age, cellular aging markers, and aneuploidy rate in in vitro fertilization (IVF) and the preimplantation genetic test for aneuploidy (PGT-A) cycles. Methods: This is a prospective cohort study recruiting 110 infertile women between August 2017 and July 2018. They were divided into young-age (<38 years, *n* = 60) and advanced-age (≥38 years, *n* = 50) groups. Peripheral leukocytes were assessed, and the granulosa cells were pooled during oocyte pickup. Mitochondrial DNA (mtDNA) copy number and telomere length (TL) were measured using real-time polymerase chain reaction. PGT-A was performed on the NGS platform. Results: mtDNA copy number and TL were positively correlated in both leukocytes (rho = 0.477, *p* < 0.001) and granulosa cells (rho = 0.361, *p* < 0.001), but the two parameters in leukocytes were not correlated with those in granulosa cells. In the young-age group, TL in the granulosa cells was the only factor correlated with the aneuploidy rate (rho = −0.283, *p* = 0.044), whereas in the advanced-age group, age was the main factor (rho = 0.358, *p* = 0.018). Conclusions: TL in the granulosa cells was negatively correlated with the aneuploidy rate in the young-age group, supporting the application of PGT-A in younger women.

## 1. Introduction

Ovarian aging, which commences around a woman’s mid- to late-thirties, is a major cause of female infertility in modern society because of delayed child bearing [1,2]. The decline in fecundability due to ovarian aging is associated with decreased oocyte quantity and quality [3]. Oocyte quantity is measured using ovarian reserve tests, including the antral follicle count and serum anti-Mullerian hormone (AMH) levels, which are used to predict the ovarian response and individualize protocol selection in assisted reproductive technology (ART) cycles [4,5,6,7,8]. Furthermore, the serum AMH level is associated with the pregnancy outcomes in ART cycles, especially in patients with advanced maternal age or decreased ovarian reserve [9,10,11,12].

Several possible mechanisms have been described to explain the decrease in oocyte quality with ovarian aging. Aneuploid oocyte [13], the consequence of meiotic error during oocyte maturation [14], is one of the major sources of declining oocyte quality. Aneuploid embryos result in implantation failure or early pregnancy loss. Therefore, in patients with advanced age and repeated implantation failure, the preimplantation genetic test for aneuploidy (PGT-A) may be used to select embryos for transfer to increase the pregnancy rate [15,16].

Another possible mechanism is changes in mitochondrial bioenergetics and biogenesis with oocyte aging [3,17]. Mitochondria play a vital role in many cellular processes, including the regulation of apoptosis, fatty acid metabolism, calcium homeostasis, balance of reactive oxygen species (ROS), and generation of adenosine triphosphate (ATP) [18]. Mitochondrial DNA (mtDNA) is circular and encodes 13 proteins of the electron transport chain [19]. mtDNA content in the oocyte decreases with oocyte aging [20]. Therefore, mtDNA copy number can be a marker of cell aging. In patients with diminished ovarian reserve (DOR), mtDNA copy number in oocytes is decreased [21,22], and cumulus cells also have low mtDNA quantity [23]. Because the evaluation of mtDNA copy number in oocytes is impractical, mtDNA copy number in the cumulus cells and granulosa cells may serve as an indirect marker of oocyte aging due to close interactions among these cells [24].

The telomere complex is located at the end of the linear chromosome. Cell division results in the shortening of telomere length (TL) to solve the end-replication problem of linear chromosomes. The increasing rate of aneuploid embryos with short TL was demonstrated in a mouse model [25]. In a human study, the short TL and low telomerase activity may be markers of primary ovarian insufficiency, a pathological form of ovarian aging [26]. Granulosa cells divide extensively and then differentiate into mural granulosa cells and cumulus cells that nurture the oocytes in growing follicles. Because granulosa cells divide many times during folliculogenesis, telomeres become increasingly shorter [27]. Therefore, the TL in granulosa cells might also be an indirect indicator of oocyte aging. In addition, our previous report indicated that TL in cumulus cells is a potential marker of oocyte competence [28].

TL is proportional to mtDNA copy number in leukocytes. Both TL and mtDNA copy number are involved in the aging process [29] and are negatively correlated with age [30,31]. TL may be a marker of the current metabolic state, whereas mtDNA copy number represents a long-term change [30]. One prospective cohort study revealed that a shorter TL in leukocytes was related to a higher aneuploidy rate in IVF patients [32]. However, there was no study analyzing the correlation among TL and mtDNA copy number with the aneuploidy rate of embryos or fetuses.

In the present study, we determined the correlation among age, cell senescence markers, and aneuploidy rate of embryos in ART cycles. We evaluated the predictive capability of TL and mtDNA copy number in granulosa cells and peripheral leukocytes for the aneuploidy rate of infertile women receiving in vitro fertilization (IVF) and PGT-A. Our findings may be valuable for counseling infertile couples about the necessity and efficacy of the PGT-A program.

## 2. Materials and Methods

### 2.1. Patient Selection

Infertile women receiving controlled ovarian hyperstimulation (COH) for the PGT-A program at the Lee Women’s Hospital, Taichung, Taiwan, were recruited between August 2017 and July 2018. Patients with stage III or IV endometriosis or ovarian failure were excluded. The indication for the PGT-A program was similar to that in our previous report, namely advanced maternal age, recurrent miscarriage, repeated implantation failure, and long-term unexplained infertility [33]. The Institutional Review Board of Chung Shan Medical University Hospital approved the study protocol (CS-17048). Written informed consent was obtained from each participant.

### 2.2. Controlled Ovarian Stimulation (COH) Protocol

The COH protocol included a standard GnRH agonist long protocol for leuprolide acetate (Lupron; Takeda Chemical Industries, Tokyo, Japan) and a standard GnRH antagonist protocol for cetrorelix acetate (Cetrotide; Merck Serono, Darmstadt, Germany), which are similar to those conducted in our previous report [33]. Follitropin alfa (Gonal-f^®^; Merck Sereno, Darmstadt, Germany) and/or hMG (Menopur^®^; Ferring, Saint-Prex, Switzerland, or Merional^®^; IBSA, Lugano, Switzerland) were used for COH from cycle day 3 until ≥2 dominant follicles reached the threshold diameter of ≥17 mm. Recombinant human chorionic gonadotropin (hCG) (Ovidrel^®^; Merck Sereno, Darmstadt, Germany) was injected to trigger final oocyte maturation, and ovum pickup was performed after approximately 36 h.

### 2.3. Collection of Granulosa Cells and Leukocytes

The granulosa cells of individual women were collected and pooled soon after oocyte recovery. The pooled granulosa cells were separated from blood cells and follicular fluid and transferred to another dish with phosphate-buffered saline (PBS; Invitrogen Corp., Carlsbad, CA, USA). The cells were then washed three times before DNA extraction. Peripheral blood was aspirated with a 5-mL syringe on the same day before oocyte pickup, and then the leukocytes within the buffy coat were collected after centrifugation.

### 2.4. Telomere Length Evaluation

Total DNA extraction from peripheral leukocytes and collected granulose cells was conducted using a DNeasy Blood and Tissue Kit (Qiagen, Hilden, Germany) according to the manufacturer’s instructions. In brief, the buffy coat and isolated granulosa cells were lysed with lysis buffer. Then, the specimen was added to NucleoSpin tissue columns. After the addition of chaotropic salts and ethanol, DNA was bound to the silica membrane. The binding process was reversible and specific to nucleic acids. Contamination was removed by washing with different buffers. Then, the DNA was eluted in 100 μL elution buffer. The DNA concentration was determined using NanoDrop 2000 (Thermo Scientific).

The telomere length in peripheral blood cells and collected granulose cells was measured with a modified real-time quantitative polymerase chain reaction (qPCR) protocol described by Cawthon [34], which was similar to that in our previous report [28]. All the amplifications were performed using SYBR Green PCR Master Mix (Applied Biosystems, Foster City, CA, USA). The reactions were performed in the Applied Biosystems Step One Plus Real-Time system (Applied Biosystems). The primers for telomere PCR were Tel1 (5′-GGTTTTTGAGGGTGAGGGTGAGGGTGAGGGTGAGGGT-3′) and Tel2 (5′-TCCCGACTATCCCTATCCCTATCCCTATCCCTATCCCTA-3′). The primers for the single-copy gene (36B4) PCR were 36B4-F (5′-CAGCAAGTGGGAAGGTGTAATCC-3′) and 36B4-R (5′-CCCATTCTATCATCAACGGGTACAA-3′). PCR cycles for telomere comprised reactions at 94 °C for 1 min, followed by 30 cycles at 95 °C for 15 s and 56 °C for 1 min. For the 36B4 gene, PCR cycles were conducted at 94 °C for 1 min, followed by 30 cycles at 95 °C for 15 s, 56 °C for 20 s, and 72 °C for 20 s. Telomere length was quantified as the relative T/S (T = telomere, S = single-copy gene) ratio.

### 2.5. Quantification of mtDNA Copy Number

The mean mtDNA copy number in peripheral leukocytes and collected granulosa cells was determined using qPCR. First, 100 ng of each DNA sample was amplified in a final volume of 25 µL containing 1 × TaqMan^®^ Universal PCR Master Mix (Applied Biosystems). Amplification was performed using the Step One Plus Real-Time system (Applied Biosystems). PCR cycles were conducted at 95 °C for 10 min, followed by 40 cycles at 94 °C for 15 s and 60 °C for 1 min. During thermal cycling, the raw fluorescence data from each sample were verified and analyzed to obtain threshold cycle (Ct) values for triple repeats of each sample. Primers specific to the unique region of the mitochondrial genome RNR1 (Hs02596859, Applied Biosystems) and the endogenous control genome β2-microglobulin (B2M) (Hs99999907, Applied Biosystems) were used for the quantification of mtDNA through qPCR. ΔCt for all samples was calculated by subtracting the average B2M Ct value from the average mtDNA Ct value (ΔCt = mtDNA Ct – B2M Ct). The content of mtDNA was calculated as relative values using the formula 2^−ΔCt^ method.

### 2.6. ART and Embryo Biopsy

Oocyte pickup, in vitro fertilization, and subsequent embryo culture, biopsy, vitrification, or thawing were performed following the approaches in our previous report [33]. In short, oocytes and embryos were cultured in a tri-gas incubator (5% O_2_, 5% CO_2_ and 90% N_2_) at 37 °C. After the fertilization of oocytes through conventional insemination or intracytoplasmic sperm injection (ICSI), individual embryos were transferred into a culture dish containing equilibrated cleavage medium (SAGE Biopharma, USA) in the tri-gas incubator. The culture medium was changed to equilibrated blastocyst medium (SAGE Biopharma) at 70–72 h after fertilization.

Only expanded blastocysts (i.e., grades AA, AB, AC, BA, BB, BC, CA, and CB based upon the Gardner grading system [35]) were selected for trophectoderm biopsy. Hence, 5–10 trophectoderm cells detached from the zona pellucida were smoothly aspirated. The biopsied trophectoderm cells were transferred into new droplets of phosphate-buffered saline and rinsed several times before their transfer into an RNAse–DNAse-free PCR tube. We moved the blastocysts back to the tri-gas incubator for at least 3 h and then used the Cryotech vitrification method (Cryotech, Japan) for the vitrification of the blastocysts.

### 2.7. Examination of Diploid–Aneuploid Levels through Next Generation Sequencing

The PGT-A protocol was similar to our previous report [33]. In brief, genomic DNA was extracted and amplified using the SurePlex DNA Amplification System (Illumina, San Diego, CA, USA). The amplified DNA product was used to prepare the genomic DNA libraries according to the VeriSeq PGS workflow (Illumina, USA). BlueFuse Multi Software (Illumina, USA) was used for data analysis, and the diploid–aneuploid levels of each sample were examined by at least two technicians. Based upon diploid–aneuploid mosaic ratios measured using the hr-NGS platform for biopsied cells [36,37,38], blastocysts were classified into the following three groups: (i) euploid blastocysts with mosaicism levels ≤ 20%; (ii) mosaic blastocysts with mosaicism levels between 20% and 50%; and (iii) aneuploid blastocysts with mosaicism levels > 50%.

### 2.8. Statistical Analysis

SPSS (v 20.0; IBM Corporation, Armonk, NY, USA) was used for data analysis. For all analyses, *p* < 0.05 was considered significant. The D’Agostino–Pearson omnibus K2 test revealed that the aging markers were not normally distributed. Therefore, we used nonparametric tests to determine whether differences between the groups reached statistical significance. The Mann–Whitney U test was applied to evaluate the differences in aging parameters between the groups. Spearman’s correlation test was used for examining the correlation among the ovarian aging markers, including age, TL, and mtDNA copy number, in both granulosa cells and leukocytes. A linear regression model was employed for multivariate analysis for the aneuploidy rate. The variables used in the regression model included aging markers found to be significant in the correlation test.

## 3. Results

We recruited 110 patients undergoing COH and PGT-A programs and divided them into young-age (<38 years, *n* = 60) and advanced-age groups (≥38 years, *n* = 50) (range: 26–44 years). Their demographic characteristics are summarized in Table 1. Compared with the young-age group, age, aneuploidy blastocyst number, and aneuploidy rate were significantly higher and serum AMH level, number of oocytes, good day-3 embryos, and good-quality blastocysts were significantly lower in the advanced-age group. Furthermore, no differences were noted in TL in leukocytes and granulosa cells or in mtDNA copy number in young-age and advanced-age groups. However, TL was significantly shorter and mtDNA copy number was significantly higher in granulosa cells than in leukocytes in both groups (all *p* < 0.001).

Table 2 presents the results of the correlation analysis of TL and mtDNA copy number in leukocytes and granulosa cells, age, and serum AMH level. The AMH level was significantly correlated with granulosa cell TL (Spearman correlation coefficient; rho = 0.385, *p* < 0.001) and mtDNA copy number (rho = 0.261, *p* = 0.006), but was not correlated with leukocyte TL and mtDNA copy number. Age was not associated with TL and mtDNA copy number in leukocytes or granulosa cells.

**Figure 1:**
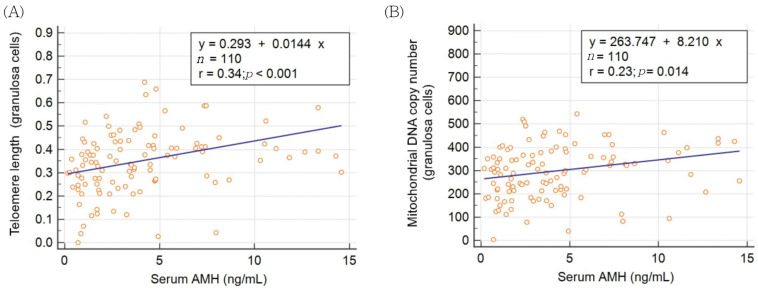
Correlation between serum anti-Mullerian hormone and (**A**) telomere length or (**B**) mitochondrial DNA copy number in granulosa cells.

The results of linear regression analysis of the AMH level, TL, and mtDNA copy number in granulosa cells are presented in Figure 1A,B. Serum AMH levels were significantly positively correlated with granulosa cell TL and mtDNA copy number.

**Figure 2:**
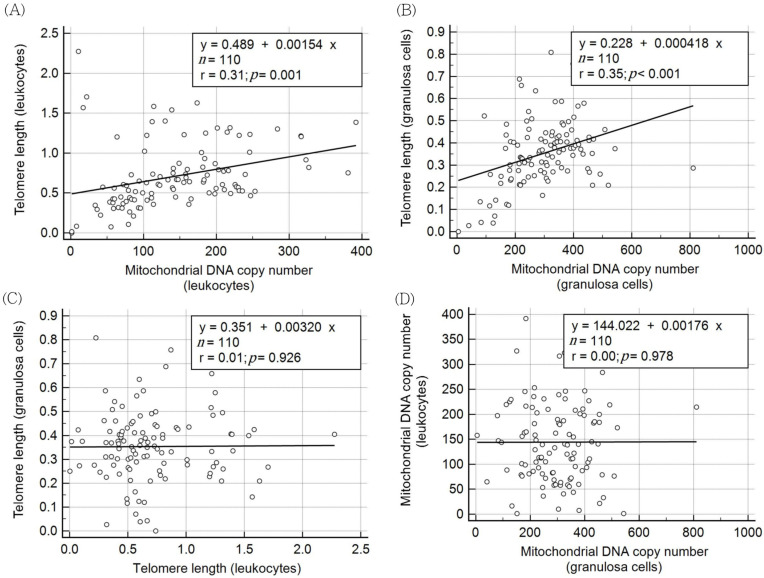
Correlation between cell senescence markers (telomere length and mitochondrial DNA copy number) in granulosa cells or leukocytes. (**A**) telomere length vs. mitochondrial DNA copy number in leukocytes. (**B**) telomere length vs. mitochondrial DNA copy number in granulosa cells. (**C**) telomere length in granulosa cells vs. leukocytes. (**D**) mitochondrial DNA copy number in granulosa cells vs. leukocytes.

Notably, TL and mtDNA copy number in leukocytes were significantly correlated (rho = 0.477, *p* < 0.001). TL and mtDNA copy number in granulosa cells were also positively correlated (rho = 0.361, *p* < 0.001). The results of linear regression analysis of TL and mtDNA copy number in the leukocytes and granulosa cells are shown in Figure 2A,B. Granulosa TL was not correlated with leukocyte TL (Figure 2C), and granulosa mtDNA copy number was not correlated with leukocyte mtDNA copy number (Figure 2D). In other words, TL and mtDNA copy number in leukocytes could not be used to estimate those in granulosa cells.

Table 3 presents the correlation between aging biomarkers and the aneuploidy rate in PGT-A cycles. The results revealed that nine of 60 patients (15%) in the young-age group and seven of 50 patients (14%) in the advanced-age group had no adequate blastocyst for trophectoderm biopsy. In the young-age group, the only significant aging biomarker of the aneuploidy rate was granulosa TL (rho = −0.283, *p* = 0.044, *n* = 51). By contrast, age (rho = 0.358, *p* = 0.018, *n* = 43) was the sole marker of the aneuploidy rate in the advanced-age group. The samples of all the patients (*n* = 94) were pooled and analyzed. Hence, age (rho = 0.496, *p* < 0.001), serum AMH (rho = −0.204, *p* = 0.049), and granulosa cell TL (rho = −0.241, *p* = 0.020) were significantly correlated with the aneuploidy rate in PGT-A cycles. Overall, multivariate linear regression analysis revealed age to be the only factor predicting the aneuploidy rate in PGT-A cycles.

**Figure 3:**
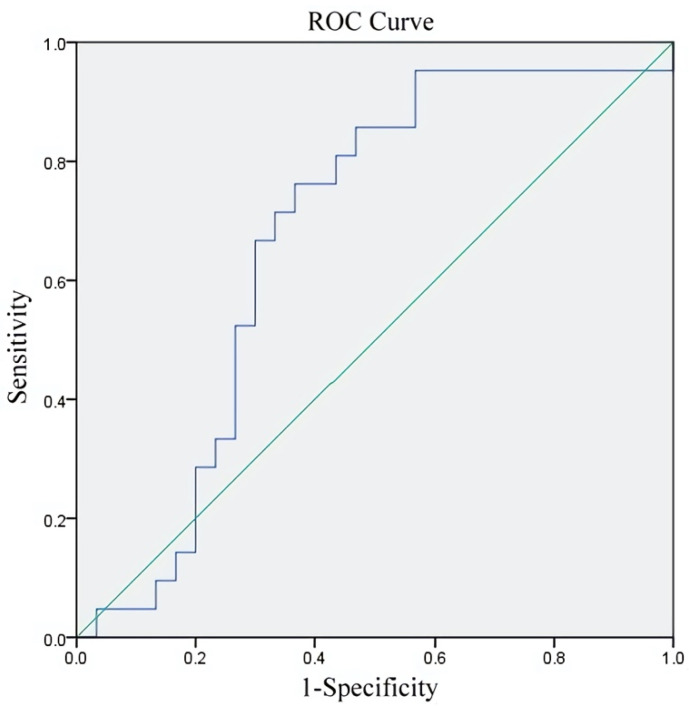
Predictive value of granulosa cell TL for aneuploidy rate in young-age group.

We calculated the areas under the respective receiver operating characteristic (ROC) curves (AUC) to confirm the predictive value of granulosa cell TL for the aneuploidy rate in the young-aged group. Figure 3 showed the AUC was 0.673 (0.521–0.825) (*p* = 0.037). The cut-off value of granulosa cell TL was 0.37 with a sensitivity of 76.2% and specificity of 63.3%.

## 4. Discussion

The TL and mtDNA copy number are excellent markers to reveal the aging of individual cells. However, the aging speed varies among various cell lineages in an individual body. The present study clearly demonstrated that serum AMH showed a higher correlation with TL and mtDNA copy number in granulosa cells compared with female age. Serum AMH is secreted by ovarian granulosa cells in adult females and is a powerful ovarian aging marker. Regarding ovarian aging, TL and mtDNA copy number in granulosa cells were closely correlated, and all these three markers may represent the aging status of the corresponding oocytes. However, neither TL nor mtDNA copy number in granulosa cells was correlated with those markers in leukocytes. The results emphasize that the aging process is different in granulosa cells and leukocytes in women.

The mtDNA copy number per cell is different, depending on their demands of energy [39], and therefore it can be a marker of mitochondrial function in a cell. Telomeres are repetitive DNA-protein complexes containing TTAGGG repeats, and the length of telomeres shortens accompanying with cell division [40]. Telomere attrition would affect the mitochondrial function and biogenesis through pathways involving the peroxisome proliferator-activated receptor gamma co-activator 1α/β (PGC-1α/β), p53, and mTORC1 [41,42]. Telomere shortening may activate the DNA damage response (DDR) which in turn causes the increase of mitochondrial content [43]. Therefore, the TL, mtDNA copy number, and cell aging are widely associated with each other, and more research is needed to clarify the mechanism.

Age tended to be negatively correlated with granulosa cell TL (*p* = 0.051) but not leukocyte TL in this study. This result may be due to the relatively small age range (26–44 years of age) and small sample size in this study. Ovarian function declines after 35–38 years of age [1]. The function of the hematopoietic system (leukocytes) and immune system declines after 45–50 years of age [44,45]. Consequently, serum AMH and granulosa cell TL may be more suitable as aging markers of oocytes than the leukocytes TL for women of reproductive age [40,46]. Although granulosa mtDNA copy number was closely correlated with granulosa cell TL and serum AMH, it was not correlated with age in the present study. Nevertheless, a prospective cohort study in 2021 revealed the relationship among leukocyte TL, age, and aneuploidy rate [32]. This discrepancy may be due to different inclusion criteria: they recruited women undergoing PGT-A cycles without a specific infertility diagnosis, whereas we included patients with specific indications for PGT-A, including advanced maternal age, recurrent miscarriage, repeated implantation failure, and long-term unexplained infertility. Further research is required to clarify the association among these aging factors.

The indication of PGT-A at the present time, although controversial, is mainly advanced maternal age, repeated implantation failure, and/or recurrent miscarriages. Nonetheless, one report indicated that PGT-A cycles may be detrimental for patients younger than 38 years at their first IVF cycles, whereas PGT-A may be beneficial for patients older than 38 years [16]. Unfortunately, there is a lack of adequate markers for these relatively young patients to predict the aneuploidy rate. Therefore, we divided our patients into two age groups (<38 and ≥38 years) to identify the parameter correlated with the aneuploidy rate in these two groups. Our results suggested that TL in granulosa cells is correlated with the aneuploidy rate in the young-age group. This may explain our previous findings that TL in cumulus cells of individual follicles is correlated with oocyte competence and the quality of ensuing embryos for patients younger than 38 years [28]. TL in granulosa cells in IVF/ICSI cycles may be a good predictor of aneuploidy, and PGT-A might be necessary for young patients with recurrent miscarriage, repeated implantation failure, and long-term unexplained infertility, depending on granulosa cell TL. The cut-off value of 0.37 for the granulosa cell TL selected by the ROC curve in this study had the greatest predictive value for the aneuploidy rate in young-age patients.

Age remained the main predictor of the aneuploidy rate in PGT-A cycles for the patients recruited into this study and the subgroup of patients with advanced age. The cause of aneuploidy is an error during meiosis of the oocyte. Human oocytes remain in the diplotene stage of meiosis I and enter meiosis II to complete the maturation process before ovulation. In addition, the granulosa cells commence proliferation and differentiation, accompanying the recruitment of follicles from the primordial follicular pool. TL and mtDNA copy number change following rapid cell division. This may explain why these two parameters in granulosa cells were not correlated with those in leukocytes.

This study has some limitations. First, the denominator in aneuploidy rate estimation was the number of biopsied blastocysts (i.e., good-quality blastocysts), and not the number of zygotes. However, our findings provide a predictive and realistic expectation of the aneuploidy rate in the PGT-A program. The results may be a good reference for counseling young patients undergoing PGT-A cycles. Second, patients with ovarian failure were excluded. This means that, for the advanced-age group, we recruited those with better ovarian reserve (high AMH level and blastocyst number) compared with women in the general population aged ≥38 years. We expected a larger difference in AMH level or blastocyst number if we compared the young-age group and advanced-age group in a nonselective way. Nonetheless, the decline in ovarian function is still prominent in advanced-age group members with relatively good ovarian reserve in the present study.

## 5. Conclusions

Serum AMH levels were positively correlated with TL and mtDNA copy number in granulosa cells, which, in turn, were significantly correlated with each other. Age was a main factor in predicting the aneuploidy rate in the advanced-age group, and TL in granulosa cells was the only marker correlated with the aneuploidy rate in the young-age group. As PGT-A might not be helpful for young patients, TL in granulosa cells may help predict aneuploidy. This finding can be used in discussions with young patients about selecting PGT-A in their ART cycle, especially for those with recurrent miscarriage, repeated implantation failure, and long-term unexplained infertility. Further studies should confirm the correlation between TL and mtDNA copy number in granulosa cells and peripheral leukocytes, as both are markers of aging and are involved in the associated mechanism of aneuploidy resulting from ovarian aging.

## Figures and Tables

**Table 1:** Demographic characteristics of the study patients. P values are determined by Mann-Whitney U test.

Group	Young (<38 y/o) *n* = 60	Advanced (≥38 y/o) *n* = 50	*p* Value
Age (year)	33.5 ± 3.1	40.7 ± 1.9	<0.001
AMH (ng/mL)	5.04 ± 3.59	3.19 ± 3.03	0.001
BMI (kg/m^2^)	23.0 ± 4.7	23.1 ± 3.4	0.501
Baseline FSH (IU/L)	6.63 ± 3.04	7.76 ± 4.12	0.144
Baseline LH (IU/L)	6.01 ± 4.68	8.06 ± 12.92	0.746
Baseline E_2_ (pg/mL)	46.3 ± 34.8	46.8 ± 41.9	0.696
Number of oocytes retrieved	16.0 ± 11.9	11.2 ± 6.4	0.017
Number of metaphase II oocytes	12.8 ± 9.8	9.1 ± 5.7	0.048
Number of 2 pronuclei (2PN)	10.0 ± 8.6	6.7 ± 4.3	0.057
Fertilization rate	74.3 ± 24.3	76.2 ± 22.6	0.653
D3 good quality embryo number	7.2 ± 6.0	4.9 ± 3.5	0.031
D5 good blastocyst number	4.4 ± 3.2 (*n* = 51)	3.3 ± 2.7 (*n* = 43)	0.044
Aneuploidy number	0.9 ± 0.9 (*n* = 51)	1.8 ± 1.5 (*n* = 43)	0.001
Aneuploidy rate (%)	23.2 ± 28.6 (*n* = 51)	61.1 ± 39.1 (*n* = 43)	<0.001
TL in leukocytes	0.76 ± 0.43 ^a^	0.66 ± 0.37 ^b^	0.214
TL in granulosa cells	0.37 ± 0.16 ^a^	0.33 ± 0.12 ^b^	0.094
mtDNA copy number in leukocytes	143.01 ± 76.5 ^c^	146.4 ± 89.26 ^d^	0.83
mtDNA copy number in granulosa cells	306.47 ± 137.2 ^c^	288.3 ± 100.85 ^d^	0.439

^a^*p* < 0.001; ^b^ *p* < 0.001; ^c^ *p* < 0.001; ^d^ *p* < 0.001.

**Table 2:** Correlation between biomarkers of aging (Spearman correlation coefficient). mt denotes mitochondria.

Spearman Correlation Coefficient	Leukocyte TL	Granulosa Cell TL	Leukocyte mtDNA Copy Number	Granulosa Cell mtDNA Copy Number
Age	−0.093	−0.186	−0.069	−0.019
	*p* = 0.334	*p* = 0.051	*p* = 0.472	*p* = 0.846
AMH	−0.015	0.385 ***	0.006	0.261 **
	*p* = 0.875	*p* < 0.001	*p* = 0.954	*p* = 0.006

**, *p* < 0.01. ***, *p* < 0.001.

**Table 3:** The correlation of aging biomarkers with aneuploidy rates (Spearman correlation coefficient) in the ART cycles with PGT-A.

Group	Young (<38 y/o) *n* = 51	Advanced (≥38) *n* = 43	Total *n* = 94
Age	0.145	0.358 *	0.496 ***
	*p* = 0.308	*p* = 0.018	*p* < 0.001
AMH	0.015	−0.263	−0.204 *
	*p* = 0.915	*p* = 0.088	*p* = 0.049
Leukocyte TL	0.222	0.166	0.139
	*p* = 0.118	*p* = 0.287	*p* = 0.182
Granulosa cell TL	−0.283 *	−0.093	−0.241 *
	*p* = 0.044	*p* = 0.552	*p* = 0.020
Leukocyte mtDNA copy number	−0.156	−0.060	−0.118
	*p* = 0.275	*p* = 0.702	*p* = 0.256
Granulosa cell mtDNA copy number	−0.162	−0.193	−0.140
	*p* = 0.255	*p* = 0.216	*p* = 0.180

*, *p* < 0.05. ***, *p* < 0.001.

## Data Availability

The data presented in this study are available on request from the corresponding author.
